# Crowding effects on the dynamics of COVID-19 mathematical model

**DOI:** 10.1186/s13662-020-03137-3

**Published:** 2020-12-01

**Authors:** Zizhen Zhang, Anwar Zeb, Ebraheem Alzahrani, Sohail Iqbal

**Affiliations:** 1 School of Management Science and Engineering, University of Finance and Economics, Bengbu, 233030 China; 2 Department of Mathematics, COMSATS University Islamabad, Abbottabad, Pakistan; 3 Department of Mathematics, Faculty of Science, King Abdulaziz University, 21589 Jeddah, Saudi Arabia; 4 Department of Mathematics, COMSATS University Islamabad, Islamabad, Pakistan

**Keywords:** Mathematical COVID-19 model, Nonlinear incidence rate, Reproduction number, Stability analysis, Nonstandard finite difference scheme

## Abstract

A disastrous coronavirus, which infects a normal person through droplets of infected person, has a route that is usually by mouth, eyes, nose or hands. These contact routes make it very dangerous as no one can get rid of it. The significant factor of increasing trend in COVID19 cases is the crowding factor, which we named “crowding effects”. Modeling of this effect is highly necessary as it will help to predict the possible impact on the overall population. The nonlinear incidence rate is the best approach to modeling this effect. At the first step, the model is formulated by using a nonlinear incidence rate with inclusion of the crowding effect, then its positivity and proposed boundedness will be addressed leading to model dynamics using the reproductive number. Then to get the graphical results a nonstandard finite difference (NSFD) scheme and fourth order Runge–Kutta (RK4) method are applied.

## Introduction

Coronaviruses cause a common group of infections that results in common cold type symptoms being one of the old classes of viruses in human history, but COVID19 became most disastrous, resulting in the highest death tolls in its track records. In history it became deadly in the shape of Serious Intense Respiratory Conditions (SARS) and Middle East Respiratory Disorder (MERS). The Severe Acute Respiratory Syndrome Coronavirus 2 (SARS-CoV-2) opened new doors for researchers and on February 12, 2020, it was formally named Novel Coronavirus disease 2019 (COVID-19) by the World Health Organization (WHO). The spread mechanism is very simple: when somebody infected sneezes or coughs, the droplets enter nearby people and soon enter their bodies through contact routes like mouth, nose, and later their lungs, and start damaging their respiratory system as it is operated through the lungs. Later it was claimed by researchers that contacting infected surfaces also causes transmission of the virus. There are many researchers stating the possible lifetime of virus on different surfaces; WHO also gave many guidelines on that. The National Institute of Health in our country states that the virus that causes coronavirus illness 2019 (COVID-19) is stable for many hours to days in aerosols and on surfaces. So far researchers are giving different time frames for the life of the virus on different surfaces. The patients are categorized in three types in start by WHO. (i) Suspect case: These are the patients with acute respiratory illness and who have been in contact with a COVID19 infected person. (ii) Exposed case: These are the patients who are infected but so far showing no symptoms of COVID19. (iii) Confirmed case: These are infectious people with authentic laboratory assertion with all the symptoms, and they are put in isolation to protect other people and the community.

Measuring the spread of a disease is a significant factor in epidemic models, which is determined by incidence rates. Generally, it is thought of as the newly infected rate per unit of time. For this purpose, $\beta SI$ with an extension of $\beta SI/(1+\alpha I)$ are used. The saturated nonlinear incidence $Sf(I)$ approach was used by Capasso and Serio [1] in the case of a cholera epidemic model. It is commonly observed in most communicable diseases that the acquired immunity may disappear after some time such as with pertussis, influenza, malaria and cholera (time may vary from disease to disease) and this puts individuals at risk again; see [2, 3]. It is also reported that in some situations the recovered patients may get mixed with a susceptible group of people with the belief of having a transient antibody [4, 5]. The SIRS model designed by Chen, having a standard incident, disease related standard incidence and death, and we have transference from the infected group to the susceptible class. So far, many research domains are in process to address the current pandemic using different mathematical models like differential equations (in integer and fractional form) (see [6–25] and the references therein).

Getting motivation from the above work, in this work a new approach is used to address COVID19 by taking the “crowding impact” in considerations. The theme behind this work is how crowding of infected individuals affects the susceptible class or population. In mathematical models this effect is addressed by a nonlinear incidence rate.

The structure of paper is as follows: in Sect. 2, we will show the proposed model, Sect. 3 is the qualitative analysis whereas Sect. 4 is all about the numerical solution of the proposed model. In the last section our conclusion and future work will be presented.

## Model formulation

In order to illustrate the crowding effects in a COVID-19 mathematical model, we proposed a model with recruitment rate *μN* for the susceptible individual and nonlinear incidence rate $\frac{\beta SI}{1+\alpha I}$. The complete dynamics is given by

1 $$\begin{aligned} \textstyle\begin{cases} \frac{dS}{dt} = \mu N-\frac{\beta SI}{1+\alpha I}-\mu S, \\ \frac{dI}{dt} =\frac{\beta SI}{1+\alpha I}-(\gamma +\mu )I, \\ \frac{dR}{dt} = \gamma I-\mu R, \end{cases}\displaystyle \end{aligned}$$

where the infection force of the disease is expressed by *βI*, the crowding effect by $\frac{1}{1+\alpha I}$, the susceptible population by $S(t)$, the infectious population by $I(t)$ and the recovered population by $R(t)$ at time t. Furthermore, *μ* is the death rate, *γ* is the recovery rate and *β* is the transmission coefficient. $N(t)$ is the total constant population,

2 $$\begin{aligned} N(t)=S(t)+I(t)+R(t), \end{aligned}$$

with

3 $$\begin{aligned} N(0)=S(0)+I(0)+R(0). \end{aligned}$$

Furthermore, from

$$\begin{aligned} S(0)\geq 0,\qquad I(0)\geq 0,\qquad R(0) \geq 0, \end{aligned}$$

we have

4 $$\begin{aligned} S(t)\geq 0,\qquad I(t)\geq 0,\qquad R(t) \geq 0. \end{aligned}$$

So the solution possesses the property of positivity.

## Dynamics of the model

### Basic properties

#### Theorem 1

(Positivity)

*Suppose that the related solution of the initial data* $(S(0), I(0), R(0))\in \mathbf{R}_{+}^{3}$ *is* $(S(t), I(t), R(t) )$. *Then for model* (1) *the positively invariant set is* $\mathbf{R}^{3}_{+}$.

#### Proof

By setting $\Lambda =\mu N$ and $\lambda _{1}=\frac{\beta I}{1+\alpha I}$, the first equation of system (1) implies that

$$\begin{aligned} \frac{dS}{dt}=\Lambda -\lambda _{1}S-\mu S. \end{aligned}$$

Suppose that the solution exists of system (1) for a certain interval $J\in [0;+\infty [$, then the above equation can be solved, for all $t\in J$, as

$$\begin{aligned} & \frac{dS}{dt}+(\lambda _{1}+\mu )S= \Lambda, \\ &\frac{d}{dt} \bigl(S(t)e^{\mu t+\int _{0}^{t}\lambda _{1}(s)\,ds} \bigr) \geq \Lambda e^{\mu t+\int _{0}^{t}\lambda _{1}(s)\,ds}, \end{aligned}$$

which implies that

$$\begin{aligned} & S(t)e^{\mu t+\int _{0}^{t}\lambda _{1}(s)\,ds}-S(0)\geq \int _{0}^{t} \Lambda e^{\mu t+\int _{0}^{w}\lambda _{1}(u)\,du}\,dw, \\ &S(t)e^{\mu t+\int _{0}^{t}\lambda _{1}(s)\,ds}\geq S(0)+ \int _{0}^{t} \Lambda e^{\mu w+\int _{0}^{w}\lambda _{1}(u)\,du}\,dw, \\ &S(t)\geq S(0)e^{-\mu t-\int _{0}^{t}\lambda _{1}(s)\,ds}+e^{-\mu t- \int _{0}^{t}\lambda _{1}(s)\,ds}\times \int _{0}^{t}\Lambda e^{\mu t+ \int _{0}^{w}\lambda _{1}(u)\,du}\,dw>0. \end{aligned}$$

Hence, $\forall t\in J$, S(t) is positive. Now the second equation of the model (1) implicitly shows that

$$\begin{aligned} \frac{dI}{dt} =\frac{\beta SI}{1+\alpha I}-(\gamma +\mu )I\geq -( \gamma +\mu )I, \end{aligned}$$

or

$$\begin{aligned} \frac{dI}{dt}\geq -(\gamma +\mu )I, \end{aligned}$$

which can be written as

$$\begin{aligned} \frac{dI}{I}\geq -(\gamma +\mu )\,dt\quad (I\neq 0). \end{aligned}$$

By integrating, we get

$$\begin{aligned} &\ln {I}\geq -(\gamma +\mu )t+C, \\ &I\geq e^{-(\gamma +\mu )t+C}, \\ &I\geq C_{1}e^{-(\gamma +\mu )t}, \end{aligned}$$

at $t=0$,

$$\begin{aligned} I\geq I(0)e^{-(\gamma +\mu )0}\geq 0. \end{aligned}$$

Therefor, for all values of *t*, $I(t)$ is positive. Similarly, the last equation of system (1) implies that

$$\begin{aligned} \frac{dR}{dt} =\gamma I-\mu R\geq -\mu R, \end{aligned}$$

or

$$\begin{aligned} \frac{dR}{dt}\geq -\mu R, \end{aligned}$$

which can be written as

$$\begin{aligned} \frac{dR}{R}\geq -\mu dt \quad(R\neq 0). \end{aligned}$$

By integrating, we get

$$\begin{aligned} &\ln {R}\geq -\mu t+K, \\ &R\geq e^{-\mu t+K}, \\ &R\geq K_{1}e^{-\mu t}, \end{aligned}$$

at $t=0$,

$$\begin{aligned} R\geq R(0)e^{-\mu 0}\geq 0. \end{aligned}$$

Hence, $R(t)$ also is positive in the given interval. □

### Existence and uniqueness of the solution

The first-order ODE in general form is

5 $$\begin{aligned} \acute{z}=g(t,z),\qquad z(t_{0})=z_{0}. \end{aligned}$$

With the help of the theorem below, we can establish the existence and uniqueness of the solution for the considered model.

#### Theorem 2

(Uniqueness of solution)

*Let us use D to denote the domain*

6 $$\begin{aligned} \vert t-t_{0} \vert \leq a,\qquad \vert z-z_{0} \vert \leq b,\quad z=(z_{1},z_{2}, \ldots,z_{n}), z_{0}=(z_{10},z_{20}, \ldots,z_{n0}), \end{aligned}$$

*and assume that the Lipschitz condition is satisfied by* $h(t,z)$:

7 $$\begin{aligned} \bigl\Vert h (t, z_{1}) - h(t, z_{2}) \bigr\Vert \leq c \Vert z_{1}- z_{2} \Vert , \end{aligned}$$

*and the two pairs* $(t, z_{1})$ *and* $(t, z_{2})$ *are in* *D*, *where* *c* *is a positive constant*. *Then there exists a constant* $\delta > 0$ *such that for the interval* $|t-t_{0}|\leq \delta $ *there exists a unique continuous vector solution* $z(t)$ *of the system* (5). *It should be noticed that condition* (6) *is satisfied with*

8 $$\begin{aligned} \frac{\partial h_{i}}{\partial z_{j}},\quad i,j=1,2,\ldots,n, \end{aligned}$$

*in the domain D*, *being continuous and bounded*.

#### Lemma 1

*If the continuous partial derivative of* $h (t, z)$ (*i*.*e*., $\frac{\partial h_{i}}{\partial z_{j}}$) *exists for a bounded closed convex domain* ℜ, *then*, *for* ℜ, *it satisfies a Lipschitz condition*. *We are interested in the domain*

9 $$\begin{aligned} 1\leq \epsilon \leq \Re. \end{aligned}$$

*Hence*, *a solution in the form of condition* (10) *is searched*:

10 $$\begin{aligned} 0< \Re < \infty. \end{aligned}$$

Now the existence theorem can be proved as follows.

#### Theorem 3

*Assume D represents the domain of* (6) *in such a manner that* (7) *and* (8) *hold*. *Then the bounded solution in domain D of* (1) *exists*.

#### Proof

Let

11 $$\begin{aligned} & h_{1}=\mu N-\frac{\beta SI}{1+\alpha I}-\mu S, \end{aligned}$$

12 $$\begin{aligned} & h_{2}=\frac{\beta SI}{1+\alpha I}-(\gamma +\mu )I, \end{aligned}$$

13 $$\begin{aligned} &h_{3}=\gamma I-\mu R. \end{aligned}$$

To show that $\frac{\partial h_{i}}{\partial z_{j}}$ $j = 1, 2, 3$ are continuous and bounded, the following partial derivatives for the proposed model are performed. By taking the partial derivative of Eq. (11) we have

$$\begin{aligned} & \frac{\partial h_{1}}{\partial S}=- \frac{\beta I}{1+\alpha I}-\mu, \\ &\biggl\vert \frac{\partial h_{1}}{\partial S} \biggr\vert = \biggl\vert - \frac{\beta I}{1+\alpha I}-\mu \biggr\vert < \infty, \\ & \frac{\partial h_{1}}{\partial I}=- \frac{\beta S}{(1+\alpha I)^{2}} \\ &\biggl\vert \frac{\partial h_{1}}{\partial I} \biggr\vert = \biggl\vert - \frac{\beta S}{(1+\alpha I)^{2}} \biggr\vert < \infty, \end{aligned}$$

and

$$\begin{aligned} &\frac{\partial h_{1}}{\partial R}=0, \\ &\biggl\vert \frac{\partial h_{1}}{\partial R} \biggr\vert = \vert 0 \vert < \infty. \end{aligned}$$

For class *I*, from Eq. (12)

$$\begin{aligned} & \frac{\partial h_{2}}{\partial S}=\frac{\beta I}{1+\alpha I} , \\ &\biggl\vert \frac{\partial h_{2}}{\partial S} \biggr\vert =\biggl\vert \frac{\beta I}{1+\alpha I} \biggr\vert < \infty, \\ & \frac{\partial h_{2}}{\partial I}=\frac{\beta S}{(1+\alpha I)^{2}}-( \gamma +\mu ), \\ &\biggl\vert \frac{\partial h_{2}}{\partial I} \biggr\vert = \biggl\vert \frac{\beta S}{(1+\alpha I)^{2}}-(\gamma +\mu ) \biggr\vert < \infty, \end{aligned}$$

and

$$\begin{aligned} & \frac{\partial h_{2}}{\partial R}=0, \\ &\biggl\vert \frac{\partial h_{2}}{\partial R} \biggr\vert = \vert 0 \vert < \infty. \end{aligned}$$

Similarly, for class *R*, from Eq. (13)

$$\begin{aligned} & \frac{\partial h_{3}}{\partial S}=0, \\ &\biggl\vert \frac{\partial h_{3}}{\partial S} \biggr\vert = \vert 0 \vert < \infty, \\ & \frac{\partial h_{3}}{\partial I}=\gamma, \\ &\biggl\vert \frac{\partial h_{3}}{\partial I} \biggr\vert = \vert \gamma \vert < \infty, \end{aligned}$$

and

$$\begin{aligned} & \frac{\partial h_{3}}{\partial R}=-\mu, \\ &\biggl\vert \frac{\partial h_{3}}{\partial R} \biggr\vert =\vert -\mu \vert < \infty, \end{aligned}$$

therefore, it can be concluded that all partial derivatives are bounded in the considered domain and are continuous. Hence, from Theorem 2, it is proved that there exists a unique solution of system (1) in D. □

### Equilibria and the reproduction number

For finding the equilibrium points of model (1), we take the algebraic system

14 $$\begin{aligned} \begin{aligned} & \mu N-\frac{\beta SI}{1+\alpha I}-\mu S = 0, \\ &\frac{\beta SI}{1+\alpha I}-(\gamma +\mu )I = 0, \\ &\gamma I-\mu R = 0.\end{aligned} \end{aligned}$$

Two solutions of the system (1) are obtained by some algebraic manipulations, one is $D_{0} = (N, 0, 0)$, a disease free equilibrium (DFE) point and the second will be discussed after computing the reproduction number of the model (1). The reproductive number is computed with the help of the next generation matrix approach presented by van den Driessche and Watmough [26].

Let $x = (I, S)$ and rewrite the model (1) for the susceptible and infected classes in the general form

15 $$\begin{aligned} \frac{dx}{dt} = \mathcal{F}(x) - \mathcal{V}(x), \end{aligned}$$

where

16 $$\begin{aligned} \mathcal{F}(x) = \begin{pmatrix} \frac{\beta SI}{1+\alpha I} \\ 0 \end{pmatrix}, \qquad\mathcal{V}(x) = \begin{pmatrix} (\gamma +\mu )I \\ -\mu N+\frac{\beta SI}{1+\alpha I}+\mu S \end{pmatrix}. \end{aligned}$$

Now the Jacobian of $\mathcal{F}(x)$ and $\mathcal{V}(x)$ of the disease free equilibrium point is

17 $$\begin{aligned} F = \begin{pmatrix} \beta N &0 \\ 0 &0 \end{pmatrix},\qquad V = \begin{pmatrix} \gamma +\mu & 0 \\ \beta N&\mu \end{pmatrix}, \end{aligned}$$

and further by using the idea of van den Driessche and Watmough [26], the reproduction number of the model (1) is follows:

18 $$\begin{aligned} \mathcal{R}_{0} = \rho \bigl(FV^{-1}\bigr) = \frac{\beta N}{\mu +\gamma }. \end{aligned}$$

#### Theorem 4

*For system* (1), *there exists a unique positive endemic equilibrium point* $D_{*}$, *if* $\mathcal{R}_{0}>1$.

#### Proof

By some algebraic manipulations, the second solution of the system (14) yields

$$\begin{aligned} &S_{*}=\frac{\mu \alpha N+\mu +\gamma }{\beta +\alpha \mu }, \\ &I_{*}=\frac{\mu (\mathcal{R}_{0} -1)}{\beta +\alpha \mu }, \\ &R_{*}=\frac{\gamma (\mathcal{R}_{0} -1)}{\beta +\alpha \mu }. \end{aligned}$$

It is clear from the values of $I_{*}$ and $R_{*}$ that is there exists a unique positive endemic equilibrium point $D_{*}$, if $\mathcal{R}_{0}>1$. □

### Stability analysis of the model

#### Theorem 5

*The system* (1) *is locally stable related to the virus free equilibrium point* $E_{0}$, *if* $\mathcal{R}_{0}<1$ *and unstable if* $\mathcal{R}_{0}>1$.

#### Proof

For local stability the Jacobian of system (1) is

19 $$\begin{aligned} J= \begin{pmatrix} -\mu - \frac{\beta I}{1+\alpha I}& -\frac{\beta S}{(1+\alpha I)^{2}} & 0 \\ \frac{\beta I}{1+\alpha I} & \frac{\beta S}{(1+\alpha I)^{2}}-(\mu + \gamma )& 0 \\ 0 & \gamma & -\mu \end{pmatrix}. \end{aligned}$$

At $E_{0}$, the Jacobian becomes

20 $$\begin{aligned} J(E_{0})= \begin{pmatrix} -\mu & -\beta N & 0 \\ 0 & \beta N -(\mu +\gamma )& 0 \\ 0 & \gamma & -\mu \end{pmatrix}, \end{aligned}$$

from which it follows that the eigenvalues are $\lambda _{1}= -\mu <0, \lambda _{3}= -\mu <0$ and $\lambda _{2}=\beta N -(\mu +\gamma )$, implying that $\lambda _{2}<0$, if $\mathcal{R}_{0}<1$. So the system (1) is locally stable for $\mathcal{R}_{0}<1$ and unstable for $\mathcal{R}_{0}>1$. The proof is complete. □

#### Theorem 6

*If* $\mathcal{R}_{0}<1$, *then the DFE point of the system* (1) *is globally stable*.

#### Proof

For the proof of this theorem, first we construct the Lyapunov function *L*:

21 $$\begin{aligned} L(I)=\ln {\frac{I}{I_{0}}}. \end{aligned}$$

Differentiating Eq. (21) with respect to time, we have

22 $$\begin{aligned} \begin{aligned} \frac{d}{dt}\bigl(L(I)\bigr)={}&\frac{1}{I}\frac{dI}{dt}, \\ \frac{d}{dt}\bigl(L(I)\bigr)={}&\frac{1}{I} \biggl( \frac{\beta SI}{1+\alpha I}-(\gamma +\mu )I \biggr) \\ ={}&\frac{\beta S}{1+\alpha I}-(\gamma +\mu ) \\ ={}&(\gamma +\mu ) \biggl(\frac{\beta S}{(\gamma +\mu )(1+\alpha I)}-1 \biggr) \\ \leq {}&(\gamma +\mu ) \biggl(\frac{\beta S}{\gamma +\mu }-1 \biggr) \\ ={}&(\gamma +\mu ) (\mathcal{R}_{0}-1 ) \\ \leq{} &0 \quad\text{for } \mathcal{R}_{0}< 1. \end{aligned} \end{aligned}$$

Therefore, if $\mathcal{R}_{0}<1$, then $\frac{d}{dt}(L(I))<0$, which implies that, for $\mathcal{R}_{0}<1$, the DFE point of the system (1) is globally stable. □

#### Theorem 7

*For* $\mathcal{R}_{0}>1$, *the system* (1) *at the positive endemic equilibrium point* $E_{*}$ *is locally stable*.

#### Proof

The Jacobian matrix of system (1) is

23 $$\begin{aligned} J= \begin{pmatrix} -\mu - \frac{\beta I}{1+\alpha I}& -\frac{\beta S}{(1+\alpha I)^{2}} & 0 \\ \frac{\beta I}{1+\alpha I} & \frac{\beta S}{(1+\alpha I)^{2}}-(\mu + \gamma )& 0 \\ 0 & \gamma & -\mu \end{pmatrix}. \end{aligned}$$

At $E_{*}$, the Jacobian becomes

24 $$\begin{aligned} J(E_{*})= \begin{pmatrix} -\mu - \frac{\beta I_{*}}{1+\alpha I_{*}}& - \frac{\beta S_{*}}{(1+\alpha I_{*})^{2}} & 0 \\ \frac{\beta I_{*}}{1+\alpha I_{*}} & \frac{\beta S_{*}}{(1+\alpha I_{*})^{2}}-(\mu +\gamma )& 0 \\ 0 & \gamma & -\mu \end{pmatrix}, \end{aligned}$$

which yields one eigenvalue $\lambda =-\mu $ and the characteristic equation

25 $$\begin{aligned} &\lambda ^{2}+ \biggl(\mu +\frac{\beta I_{*}}{1+\alpha I_{*}}- \frac{\beta S_{*}}{(1+\alpha I_{*})^{2}}+(\mu +\gamma ) \biggr)\lambda \\ &\quad {}+ \biggl(\mu +\frac{\beta I_{*}}{1+\alpha I_{*}} \biggr) \biggl( \frac{\beta S_{*}}{(1+\alpha I_{*})^{2}}+(\mu +\gamma ) \biggr)+ \biggl( \frac{\beta S_{*}}{(1+\alpha I_{*})^{2}} \biggr) \biggl( \frac{\beta I_{*}}{1+\alpha I_{*}} \biggr)=0. \end{aligned}$$

It is clear, for $\mathcal{R}_{0}>1$, that

$$\begin{aligned} &\biggl(\mu +\frac{\beta I_{*}}{1+\alpha I_{*}}- \frac{\beta S_{*}}{(1+\alpha I_{*})^{2}}+(\mu +\gamma ) \biggr) \\ &\quad= \biggl(\mu +\frac{\beta I_{*}}{1+\alpha I_{*}}- \frac{\gamma +\mu }{(1+\alpha I_{*})}+(\mu +\gamma ) \biggr)>0 \end{aligned}$$

and

$$\begin{aligned} \biggl(\mu +\frac{\beta I_{*}}{1+\alpha I_{*}} \biggr) \biggl( \frac{\beta S_{*}}{(1+\alpha I_{*})^{2}}+(\mu +\gamma ) \biggr)+ \biggl( \frac{\beta S_{*}}{(1+\alpha I_{*})^{2}} \biggr) \biggl( \frac{\beta I_{*}}{1+\alpha I_{*}} \biggr)>0. \end{aligned}$$

Hence, the system (1) is locally stable at $E_{*}$ for $\mathcal{R}_{0}>1$. The proof is complete. □

#### Remark 1

Although the stability analysis of $E_{*}$ is an interesting and separate mathematical problem, while for prevention of the disease one is to find an effective strategy, the main focus of this work is on the specific condition $\mathcal{R}_{0} < 1$.

### $\mathcal{R}_{0}$ sensitivity analysis

From Theorem 6 it follows that we can control the parameters such that $\mathcal{R}_{0} < 1$. This leads to the best strategy to prevent and restrain the disease. In detail, when $\mathcal{R}_{0} < 1$, then

$$\begin{aligned} \lim_{t \to \infty } S(t) = N, \qquad \lim_{t \to \infty } I(t) = \lim _{t \to \infty } R(t) = 0, \end{aligned}$$

which shows that the spreading speed of the coronavirus can be reduced and prevented in the future. Hence, a sensitivity analysis of $\mathcal{R}_{0}$ is carried out to select the influential parameters to control the rapidly spreading current pandemic.

It is easy to verify that

26 $$\begin{aligned} \begin{aligned} &\frac{\partial \mathcal{R}_{0}}{\partial \beta } = \frac{N}{\mu +\gamma } > 0, \\ &\frac{\partial \mathcal{R}_{0}}{\partial \mu } = - \frac{\beta N}{(\mu +\gamma )^{2}}< 0, \\ &\frac{\partial \mathcal{R}_{0}}{\partial \gamma } = - \frac{\beta N}{(\mu +\gamma )^{2}} < 0. \end{aligned} \end{aligned}$$

Equation (26) can be used to obtain different parameters in such a way that $\mathcal{R}_{0}$ remains less than one. Hence, necessary actions can be taken on the basis of Eq. (26) to reduce the speed of the coronavirus.

## Numerical method and results

In order to get the numerical output from model (1), the NSFD method is utilized. The solution via the NSFD method is obtained via an iteration process [27, 28]. Assume the nonstandard ODE

$$\begin{aligned} w'_{k}=f[t,w_{1},w_{2}, \ldots,w_{n}], \end{aligned}$$

where $k=1,2,,\ldots,n$, then by the NSFD method

$$\begin{aligned} & w'_{1}=\frac{w_{1,k+1}-w_{1,k}}{h}, \\ &w'_{2}=\frac{w_{2,k+1}-w_{2,k}}{h}, \\ &\cdots \\ &w'_{n}=\frac{w_{n,k+1}-w_{n,k}}{h}. \end{aligned}$$

Now, for a numerical solution of system (1) using the NSFD method, it gives the following results:

27 $$\begin{aligned} &S_{k+1}=\frac{h\mu N+S_{k}}{1+h\mu +h\beta I_{k}/(1+\alpha I_{k})}, \end{aligned}$$

28 $$\begin{aligned} &I_{k+1}= \frac{h\beta I_{k}S_{k+1}/(1+\alpha I_{k})+I_{k}}{1+h(\mu +\gamma )}, \end{aligned}$$

and

29 $$\begin{aligned} R_{k+1}=\frac{h\gamma I_{k+1}+R_{k}}{1+h\mu }. \end{aligned}$$

**Figure 1:**
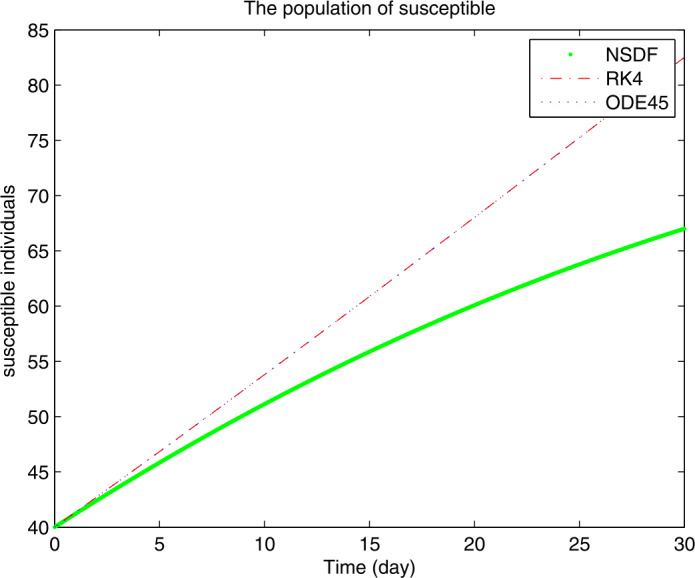
Plot for $S(t)$ versus time *t*

**Figure 2:**
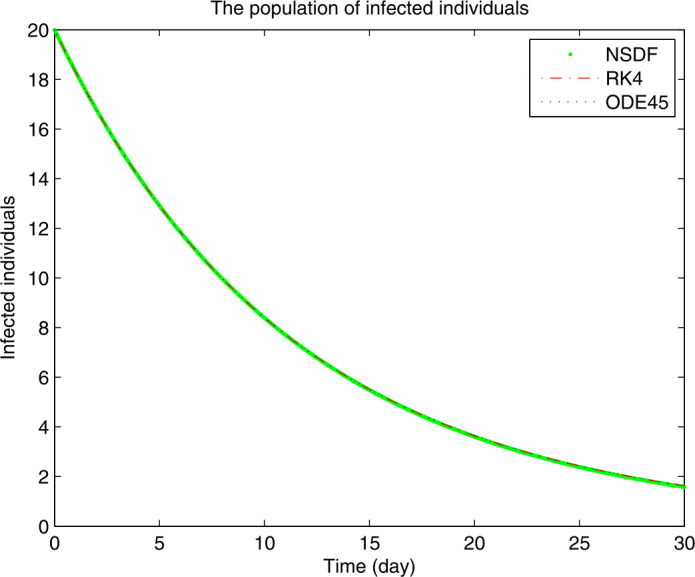
Plot for $I(t)$ versus time *t*

**Figure 3:**
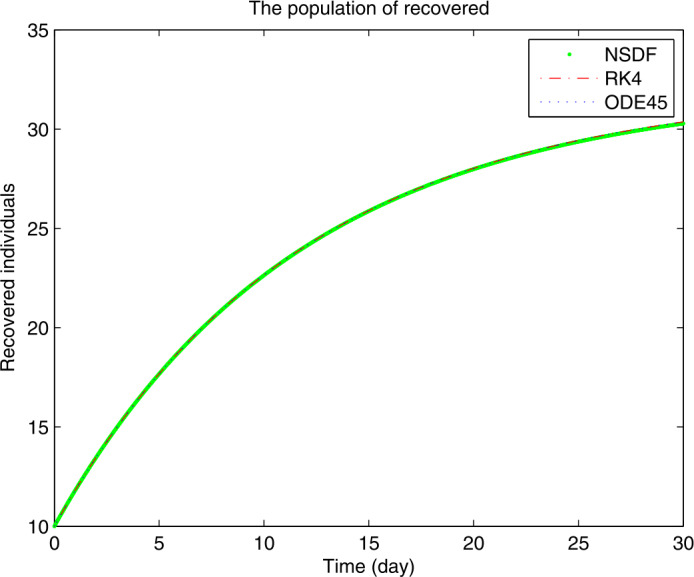
Plot for $R(t)$ versus time *t*

Figures 1–3 show the solutions for $S(t)$, $I(t)$ and $R(t)$ obtained by NSFD, RK4, and ode45 for $\mathcal{R}_{0}<1$, when the contact rate is chosen in a small range, then the spread of the current coronavirus disease may be controlled. From Fig. 1, we see how the disease controls which results are leading to an increase of the susceptible class. On the other hand as the susceptibility is increasing the infection goes to extinction reaching stability in Fig. 2. The decrease in the infection class yields increase an in the recovered class as shown in Fig. 3. For the numerical solutions, we consider the initial values $S=40, I=20, R=10$ and for the parameters values from [21], for the remaining features of the model one can use the real-life data of some specific country or of the whole world.

## Conclusion

This work presented the crowding effects of infective individuals over the susceptible population specially for the current pandemic. The crowding effect is described by a nonlinear incidence rate in the mathematical model. In this work, the formulation of the model is presented keeping in mind the crowding effect, which is in a large range of interaction of the infected population with the susceptible population. The dynamics of the model is presented based on the reproductive number and one showed the local and global stability of the proposed model. For a numerical solution we used the nonstandard finite difference (NSFD) scheme and the fourth-order Runge–Kutta (RK4) method and the obtained results are shown graphically.

## Data Availability

The authors confirm that the data supporting the findings of this study are available in the article.
